# Early Social Environment Affects the Endogenous Oxytocin System: A Review and Future Directions

**DOI:** 10.3389/fendo.2015.00032

**Published:** 2015-03-11

**Authors:** Emily Alves, Andrea Fielder, Nerelle Ghabriel, Michael Sawyer, Femke T. A. Buisman-Pijlman

**Affiliations:** ^1^School of Medical Sciences, University of Adelaide, Adelaide, SA, Australia; ^2^School of Midwifery, University of South Australia, Adelaide, SA, Australia; ^3^School of Psychology, University of South Australia, Adelaide, SA, Australia; ^4^School of Paediatrics and Reproductive Health, University of Adelaide, Adelaide, SA, Australia; ^5^Research and Evaluation Unit, Women’s and Children’s Health Network, Adelaide, SA, Australia

**Keywords:** oxytocin, early-life environment, research design, individual differences, mother–infant bonding

## Abstract

Endogenous oxytocin plays an important role in a wide range of human functions including birth, milk ejection during lactation, and facilitation of social interaction. There is increasing evidence that both variations in the oxytocin receptor (OXTR) and concentrations of oxytocin are associated with differences in these functions. The causes for the differences that have been observed in tonic and stimulated oxytocin release remain unclear. Previous reviews have suggested that across the life course, these differences may be due to individual factors, e.g., genetic variation (of the OXTR), age or sex, or be the result of early environmental influences, such as social experiences, stress, or trauma partly by inducing epigenetic changes. This review has three aims. First, we briefly discuss the endogenous oxytocin system, including physiology, development, individual differences, and function. Second, current models describing the relationship between the early life environment and the development of the oxytocin system in humans and animals are discussed. Finally, we describe research designs that can be used to investigate the effects of the early environment on the oxytocin system, identifying specific areas of research that need further attention.

## The Endogenous Oxytocin System

Oxytocin, a mammalian hormone, is a naturally produced neuropeptide with nine amino acids. Oxytocin is mainly produced in magnocellular neurons in the hypothamalic paraventricular and supraoptic nuclei (1). The magnocellular neurons release oxytocin into circulating blood via the pituitary gland, while the parvocellular oxytocin neurons release oxytocin in other areas of the central nervous system (CNS). Recent evidence has demonstrated that oxytocinergic axons ending in forebrain regions including the central amygdala and nucleus accumbens may originate exclusively in the magnocellular neurons of the paraventricular nucleus (2). Evidence also indicates that central projections of hypothalamic oxytocin neurons may be widespread, and that oxytocin release from local axonal endings may be able to control region-associated behaviors (2). Oxytocin is also produced in a number of peripheral tissues and organs, such as the uterus, ovaries, testis, vascular endothelium, and the heart (3). For a review of the anatomy and functional aspects of the oxytocin system, see Kiss and Mikkelson (4).

Oxytocin is released in response to a range of internal and external stimuli. Historically, it is known that oxytocin is released in response to vaginocervical and nipple stimulation and plays important roles in mammalian uterine contraction and lactation. Oxytocin also plays a crucial role in the milk ejection reflex. In lactating women, oxytocin peaks during the morning and then declines until the beginning of the afternoon, and it is released in a cyclic manner, with salivary oxytocin concentrations at their highest within 30 min before feeding begins (5). This indicates that the brain may be able to release oxytocin in anticipation of future behaviors and interactions.

More recent data demonstrate the pro-social role of oxytocin, including its role in social and emotional regulation (6), orgasm (7), regulating stress, and anxiety and facilitation of pair, maternal and infant bonding (8–12). Interestingly, the release of oxytocin is dependent on the social context. For example, calves more readily release oxytocin when suckling milk directly from their mother’s udder than when drinking the same milk from a bucket (13). Another example shows that oxytocin administration combined with psychological support of a friend lowered salivary cortisol concentrations and was correlated with decreased anxiety and increased calmness in human males (8).

Large individual differences exist in basal concentrations of oxytocin and in response to stimulation [for a review, see Ref. (12)]. Basal individual oxytocin concentrations in humans have been found to vary by thousands of pg/ml within the same study (14). Individual differences, such as genetic variation of the oxytocin receptor (OXTR) may explain some of the variation. Expression of the OXTR also varies in a sex-specific way. It is therefore possible that an interaction with the estrogen system plays a role that warrants further attention and investigation (15).

## Oxytocin and the Early Social Environment

The early-life environment is critical for development in humans and other mammals [for a review on the long-term impact of early-life events, see Ref. (16)]. A significant component of the early-life environment is mother–infant bonding. Social experiences in the early-life period and bonding form the basis for healthy social and emotional development, possibly of the mechanism that manages stress and resilience (11). The well-documented protective associations between secure attachments and later social functioning and behavior underscore the need to understand the origins of attachments and identify the specific individual differences that influence attachment and development (17). Current research highlights the link between oxytocin concentrations and a specific set of maternal bonding behaviors and attitudes in humans (18–24). There is evidence to suggest that oxytocin plays an important role in facilitation of mother–infant bonding [for reviews, see Ref. (10, 25)] and that the early social environment can shape the developing oxytocin system. There is substantial literature documenting the relationship between oxytocin and the early-life environment in animals (26–28); however, minimal research has focused on this area in humans. Importantly, there is only limited data about the normal development of the oxytocin system in humans over time (11).

Oxytocin plays an important role in priming mammals to form social bonds, but in turn, the early social environment may also be able to shape the development of the oxytocin system [for a review, see Ref. (11)]. Interestingly, studies have demonstrated correlations between infant and parental oxytocin concentrations and parenting behaviors in child–parent interactions (14, 29). The following section will review the influence of prenatal and postnatal environment on the oxytocin system. Additionally, maternal mental health problems will be addressed as they can negatively affect the mother–infant bond.

### Prenatal environment

In humans, prenatal stressors (including substance use, maternal depression, and chronic stress) can lead to a range of abnormal neurodevelopmental outcomes for infants [for a review, see Ref. (30)]. A recent human study found that male and female fetuses respond differentially to chronic maternal stress, indicating that sex may be an important factor influencing fetal development (31).

Lee and colleagues (32) demonstrated that a regime of prenatal stressors in pregnant rats caused numerous changes in the oxytocin systems of adult male offspring (less oxytocin mRNA in the paraventricular nucleus, but increased OXTR binding in the central amygdala). Prenatally stressed rats showed disturbed social behavior, which could be normalized with local oxytocin administration in the central amygdala. Cross fostering the pups did not normalize the behavior.

Drug abuse, including smoking, heavy consumption of alcohol, and illegal substances, can adversely affect fetal development and can also impair infant health and development. Alcohol can cause numerous growth impairments, and is especially well known for its harmful effects on the developing nervous system. Tobacco and cannabis smoking are also notorious for fetal growth-restricting and gestation-shortening effects (33). There is ample evidence on the long-term effects of prenatal drug use (ethanol, stimulants, and nicotine) on rodent offspring, focusing on social behavior and drug use and changes to the oxytocin system (33). Exposure to cocaine in the prenatal period in rats affects both the oxytocin system and social behavior and increases susceptibility to addiction later in life (34, 35).

### Postnatal environment

The oxytocin system continues to develop after birth, and this development may be critical for providing humans with the skills for healthy social functioning (25). There is extensive research focusing on the long-term effects of early-life adversity and social environment on the oxytocin system; however, most of these studies use animal models. Differences in rearing conditions and bonding behavior can influence adult social and parental behavior in prairie voles (9). Additionally, quality of maternal behavior has been linked to differences in oxytocin concentrations and OXTR expression observed in animal models (9, 36). For example, high levels of maternal licking resulted in increased plasma oxytocin concentrations in neonatal rats (37). Furthermore, Kojima and colleagues (38) found that maternal skin-to-skin contact stimulates rat pups’ central oxytocin concentrations. Early-life adversity and differences in the early social environment may also adversely affect the expression and concentration of the OXTR. Veenema (26) provides a thorough review on the effects of early-life manipulations in rodents on the distribution and expression of oxytocin and vasopressin receptors. Bales and Perkeybile (39) also provide a good review of the effect of early experience on the OXTR system.

Human studies with infants and their parents have demonstrated how oxytocin levels rise in response to social interaction and how infant and parental oxytocin concentrations correlate (39). These findings have been supported in previous research measuring cerebrospinal fluid (CSF) oxytocin concentrations, which have shown that higher infant CSF oxytocin concentrations were positively correlated with active initiation and interest in parental social interaction (40). The human oxytocin system seems to be receptive to both positive and negative early social experiences, such as separation (41). Infants who experienced high affect parent–infant synchrony (i.e., monitoring and responding) showed increase oxytocin saliva measures compared to infants reared in the presence of low affect parent–infant synchrony (42). This suggests that the early environment may directly affect peripheral oxytocin concentrations in humans. Importantly, a study by Wismer Fries and colleagues (43) reported significant social deficiencies and low oxytocin concentrations in children reared in extremely aberrant social environments. These results indicate that the early environment may influence cross-generation transmission of human social attachments and behavior. They also support the notion that peripheral oxytocin measurements may be correlated to social behavior in both infants and adults.

### Maternal anxiety, stress, and depression

Maternal mental health problems can greatly influence the mother–child bond as they affect the way they perceive their child’s needs and cues, their stress resilience, and their general emotional availability.

Research has shown that mothers with postpartum anxiety report significantly lower bonding with their infants than healthy mothers (44). Depressed mothers are more likely to perceive their infant’s behavior negatively than healthy mothers (45). Depression can also play an important role in influencing the early-life environment through its effect on maternal behavior and mother–infant bonding. Research shows that the most significant predictor of lower postnatal maternal attachment was depressive symptoms experienced during the final stages of pregnancy and in the postnatal period (18, 46). Despite this emerging evidence, a recent review of the literature (47) identifies that mechanisms underlying maternal stress, depression and anxiety, and their effects on infant outcomes are poorly understood. Due to its important role in mother–infant bonding, oxytocin could be involved in these mechanisms; however, further study is needed.

Interestingly, a recent pilot study found that exogenous administration of oxytocin stimulated protective behavior in mothers with postnatal depression (48). Eapen and colleagues (49) also found an association between lower plasma oxytocin levels in the post partum period and separation anxiety and depression during pregnancy. Further study is needed to determine the relationship between maternal depression and oxytocin, how this relationship may affect infants, and the potential role of exogenous oxytocin in treating depression.

## Considerations for Research

The previous sections have introduced the endogenous oxytocin system and its relationship to early environmental and social factors. The present section will identify limitations in the current literature and provide suggestions for future research.

This review has established that there is evidence showing that early social environment in animals is correlated with altered oxytocin concentrations. Particular attention to maternal behaviors during the postnatal period is warranted given the established link between mother–infant dyadic interactions and later physical, emotional, and social health. Studies by Feldman and colleagues (14, 18, 41, 42) have provided insight into the range of saliva and plasma oxytocin concentrations that should be expected in both mothers and babies. However, very little is still known about the normal development of the oxytocin system into childhood and adolescence.

Additionally, a proposed direction of research would be to determine how an early adverse social environment in humans affects the oxytocin system. Of interest are changes in basal concentrations, differences in reactivity of the oxytocin system under stress or social interaction and changes in OXTR characteristics (e.g., investigation of epigenetics, binding affinity, up- or downregulation of receptors, and methylation).

Up- or downregulation of the number of OXTRs, localization and sensitivity of OXTRs are currently difficult to research in humans, as there is no radio-active ligand that can be used in this process. Therefore, a number of different research techniques are needed to investigate these changes through other means, including the collection of biological samples during observational studies and psychological testing undertaken in large cohort studies, and further refinements of assay methodologies.

Epigenetic changes will be of interest to investigate when determining positive or negative effects of early social environment on the developing oxytocin system. Epigenetics refers to the regulation of DNA transcription without alteration of the original sequence. DNA methylation is an important epigenetic modification in response to, e.g., oxygen deprivation, trauma, or drug use. A recent study found that traumatic experiences and stressful life events in early life were associated with higher methylation of the NR3C1 gene (50). Although the topic of epigenetics has not been thoroughly explored in the present review, there is promising research indicating its importance (28, 51–53).

### Sample collection and measurement

Extensive discussion in the field addresses the most suitable and reliable method of collecting and analyzing oxytocin samples, both from the periphery and centrally. Different methods of sample collection and measurement contribute to the large range in oxytocin concentrations reported in the literature (54).

Human research into oxytocin is significantly more difficult than animals due to the inability to non-invasively assess brain oxytocin pathways and concentration. However, peripheral oxytocin concentrations can be tested through plasma, urine or saliva. These measures possibly may not directly correspond with brain concentrations; however, research has shown that changes in behavior are linked to changes in peripheral oxytocin concentrations (14, 54). There is also increasing evidence suggesting that peripheral oxytocin is reflective of changes in central concentrations. For example, a recent study of human children found that plasma oxytocin concentrations significantly and positively predicted CSF oxytocin concentrations (55).

Collecting samples of oxytocin from infants provide an additional challenge to researchers. CSF, urine, and plasma samples are generally unsuitable in these situations as the collection method is invasive and painful; furthermore, it is unlikely that parents will provide consent for their infant’s participation in a study with these extraction methods. Finding correlations between oxytocin concentrations in urine and social behavior has shown some success in both animal and human studies (56, 57). However, this collection method may still not be appropriate when attempting to determine an acute infant increase in oxytocin in response to a parent-infant interaction, as urine concentrations represent accumulated oxytocin concentrations and are not always readily available for collection, especially in infants. Saliva, therefore, would appear to be the most ideal method of measuring oxytocin concentrations in infants, with adoption of this method resulting in significant associations between infant and parent oxytocin concentrations and social behavior (42). Recent research from our group (unpublished) has supported previous studies, finding that oxytocin concentrations can be determined from both adult and infant saliva samples. Adults are able to provide this sample by simply spitting into a tube. Saliva from infants can be collected by collecting passive drool from the baby’s mouth with a syringe and then dispensing into a tube. Parents have also willingly provided infant consent for this form of collection as it is a minimally invasive and completely painless extraction method. A limitation of this process, however, is that some infants are more accepting of syringing than others, influencing the collection time. This process of collection could also potentially be difficult if the infant is distressed.

Further refinement of oxytocin assay methodologies is also needed. A comparison of enzyme immunoassays (EIA) and radio immunoassays (RIA) of human plasma samples with and without extraction found that without extraction, plasma measured by EIA was more than 100 times higher than in extracted plasma, and the correlation between them was minimal (*p* = 0.54) (58). The same study found that when using an RIA, the majority of samples (90%) were below the level of detection. This could provide some insight into the general inconsistency of results across different studies, indicating the need to refine oxytocin assay methodologies. Currently, comparing group differences within experiments is the most reliable approach.

Specificity of the role of oxytocin provides a challenge in determining the link with early adversity. Oxytocin and vasopressin are very similar in structure, and the two hormones have a high affinity for each other’s receptors (59). It is suggested that early social experiences influence sensitivity to both the oxytocin and vasopressin systems (28). This emphasizes the need for further understanding of the systems interacting with oxytocin and the relationship they may have with early adversity and development in humans.

While focusing on the effect of early life social experiences on infants and their developing oxytocin system, it is important to acknowledge that the oxytocin system has numerous bilateral interactions (e.g., dopamine and HPA-axis) that are likely to affect behavior [for a review, see Ref. (12)]. Changes to the oxytocin system will also influence these systems and vice versa. Additionally, systems like the HPA-axis will be affected by environmental influences as well. Researchers focusing on long-term effects of early social environments need to keep these considerations in mind and try to address all the dimensions.

### Characterization and measurement of early adversity

Another research challenge is measuring early adversity itself. First, there is little consensus on the term, and second, it is conceptualized differently across different disciplines, i.e., neglect, abuse, toxic stress, disorganized attachment, reduced bonding, etc. Detecting and quantifying early adversity is challenging when relying on questionnaires. Observing single mother–child interactions in a laboratory setting or home environment can provide great insight into the early social environment. Assessing attachment status is often not an option, as extensive training is needed. Mothers of infants who may be experiencing early adversity may be reluctant to participate in behavioral observations, although large cohort studies may be able to incorporate this in sub-groups. The next challenge is selecting a tool that can be used to score behavior in a dyad and quality of the interaction (e.g., reciprocity), which is objective, intuitive, and has large inter-rater reliability. There are few scales that have been validated for this purpose meeting all these criteria.

## Concluding Remarks

Review of the literature indicates that the early-life is a vital period for healthy development in humans, and that oxytocin is an important regulator of emotional development. Specifically, investigating the effect of early adversity on the endogenous oxytocin system is important in understanding normal behavior and social disorders. Understanding this effect can increase knowledge of how the early environment appears to change the way humans are hardwired to respond in social and stressful situations. A number of animal studies have been conducted; however, more human research specifically focusing on this relationship is necessary incorporating a large range of study designs. Future studies must consider the obstacles that prevent conducting this research, such as collecting appropriate peripheral oxytocin measures and selecting a suitable tool to score both infant and maternal behavior.

## Conflict of Interest Statement

The authors declare that the research was conducted in the absence of any commercial or financial relationships that could be construed as a potential conflict of interest.
